# Impact of SRY-Box Transcription Factor 11 Gene Polymorphisms on Oral Cancer Risk and Clinicopathologic Characteristics

**DOI:** 10.3390/ijms21124468

**Published:** 2020-06-23

**Authors:** Chia-Ming Yeh, Chiao-Wen Lin, Hsueh-Ju Lu, Chun-Yi Chuang, Chia-Hsuan Chou, Shun-Fa Yang, Mu-Kuan Chen

**Affiliations:** 1 Institute of Medicine, Chung Shan Medical University, Taichung 402, Taiwan; yehcm0525@outlook.com (C.-M.Y.); wishwing1109@hotmail.com (C.-H.C.); 2 Cancer Research Center, Changhua Christian Hospital, Changhua 500, Taiwan; 3 Department of Otorhinolaryngology-Head and Neck Surgery, Changhua Christian Hospital, Changhua 500, Taiwan; 4 Institute of Oral Sciences, Chung Shan Medical University, Taichung 402, Taiwan; cwlin@csmu.edu.tw; 5 Department of Dentistry, Chung Shan Medical University Hospital, Taichung 402, Taiwan; 6 Division of Medical Oncology, Department of Internal Medicine, Chung Shan Medical University Hospital, Taichung 402, Taiwan; hsuehju0311@gmail.com; 7 School of Medicine, Chung Shan Medical University, Taichung 402, Taiwan; cyi4602@gmail.com; 8 Department of Otolaryngology, Chung Shan Medical University Hospital, Taichung 402, Taiwan; 9 Department of Medical Research, Chung Shan Medical University Hospital, Taichung 402, Taiwan

**Keywords:** SOX11, oral squamous cell carcinoma, single nucleotide polymorphisms, metastasis

## Abstract

Oral cancer is among the most common cancers worldwide and has become a major global health problem because of its relatively high morbidity and mortality rates. The sex-determining region on the Y-chromosome-related high-mobility-group box (SOX) transcription factor 11 (SOX11) plays a key role in human development and differentiation and is frequently increased in various human cancers. However, the clinical significance of SOX11 polymorphisms in oral cancer and their association with oral cancer risk are unclear. In this study, we included 1196 patients with oral cancer and 1200 controls. Real-time polymerase chain reaction was applied to analyze three SOX11 single-nucleotide polymorphisms (rs77996007, rs66465560, and rs68114586). Our results shown that SOX11 polymorphisms carriers with betel quid chewing were found to have an 8.38- to 9.23-fold risk to have oral cancer compared to SOX11 wild-type carriers without betel quid chewing. Furthermore, oral cancer patients who carried SOX11 rs77996007 “TC + CC” variants were significantly associated with large tumor size (AOR, 1.324; 95% CI, 1.047–1.674; *p* = 0.019). Moreover, a database analysis using the Cancer Genome Atlas suggested that SOX11 mRNA expression was high during the tumor development process. In conclusion, our results suggest that SOX11 rs77996007 is involved in oral cancer progression and clinical characteristics.

## 1. Introduction

Oral and pharyngeal cancer has an annual incidence of approximately 5 million and causes approximately 25,000 deaths each year in the United States. Cancers of the oral cavity and the pharynx are among the most common cancers in the world. Nearly 53,000 new cases of oral and pharyngeal cancer and 10,860 deaths were recorded in 2019 worldwide, with a wide geographical variation in incidence being reported [1]. More than 90% of oral cancer cases involve oral squamous cell carcinoma (OSCC), which has become a global health problem because of its relatively high morbidity and mortality rates [2]. Surgery and chemoradiotherapy are the main treatments for oral cancer. Despite considerable progress in the treatment of oral cancer, the 5-year survival rate for advanced (stage III and IV) oral cancer is approximately 20% [3]. Tobacco and alcohol are the main risk factors for oral cancer. The risk of oral cancer increases when both factors are present. Exposure to one or both factors can explain approximately 70% of cancers in this region [4,5].

The sex-determining region on the Y-chromosome-related high-mobility-group box (SOX) transcription factor family contains more than 20 members in vertebrates; these members are divided into eight groups, spanning from SOXA to SOXH [6,7,8]. These genes originated in a series of evolutionary processes, including replication and divergence [8,9], and were identified more than 20 years ago. Since then, numerous studies have demonstrated their fundamental and dynamic functions in embryonic development and diseases [7,10]. SOX transcription factors play a major role in regulating stem cell maintenance and terminal differentiation of different cell types [11]. SOX11 belongs to the SOXC group, which also includes SOX4 and SOX12 [12]. SOX11 plays a key role in human development and differentiation. *Sox11*-deficient mice die of cardiac artery outflow tract malformations, suggesting that *Sox11* has a vital function in tissue remodeling [13]. Recent studies have shown that *SOX11* messenger RNA (mRNA) is frequently increased in various human cancers, including breast cancer [14], mantle cell lymphoma [15], gastrointestinal cancer [16], epithelial ovarian cancer [17], and nervous system neoplasms. *SOX11* upregulation promotes epithelial-to-mesenchymal transition in breast cancer cells [18]. A single-nucleotide polymorphism (SNP) is a common genetic variation that can affect protein expression and structure and lead to disease susceptibility [19]. The genetic polymorphisms of SOX transcription factors have been associated with the risk of numerous cancer types, including gallbladder cancer [20], breast cancer [21], and endometrial cancer [22]. Moreover, recent studies have reported that both the SOX11 gene and protein expression were significantly overexpressed in recurrent oral cancer tissues and may promote oral cancer invasion and progression [23,24]. However, no studies have explored the correlation between *SOX11* SNPs and oral cancer. 

Accordingly, we conducted this case–control study to investigate the relationship between three SOX11 gene polymorphisms (rs77996007, rs66465560, and rs68114586) and clinical pathological characteristics of oral cancer patients in order to identify patients at an increased risk of oral cancer and provide a theoretical basis for further clinical prevention of oral cancer.

## 2. Results

### 2.1. Characteristics of Study Participants

This study included 2396 participants, and Table 1 presents descriptive statistics for the demographic characteristics of the participants. The participants comprised 1196 patients with OSCC (case group) and 1200 controls (control group). The results revealed a statistically significant difference in betel chewing (*p* < 0.001), cigarette smoking (*p* < 0.001), and alcohol drinking (*p* < 0.001) between the case and control groups. However, differences in age distribution between the two groups were not statistically significant (*p* = 0.966).

### 2.2. Association of SOX11 Polymorphisms with Oral Cancer Risk

To investigate the association between the allele frequency of SOX11 SNPs and the risk of oral cancer, the allele frequencies of SOX11 rs77996007, rs66465560, and rs68114586 in the case and control groups were tested (Table 2). 

The allele frequencies of SOX11 rs77996007, rs66465560, and rs68114586 were predominantly distributed in the controls and patients with oral cancer heterozygous for TC, homozygous for TT, and homozygous for Ins/Ins. Patients who carried SOX11 rs77996007 TC were at a higher risk of oral cancer compared with the controls (OR = 1.218; 95% CI = 1.026–1.446). However, after adjustment for age, betel quid chewing, cigarette smoking, and alcohol drinking, no significant difference in distributions of SOX11 SNPs rs77996007, rs66465560, or rs68114586 was observed between the case and control groups (Appendix A, Table A1).

### 2.3. Association of SOX11 SNPs with Oral Cancer Risk Considering Betel Quid Chewing

Table 3 presents the associations between environmental risk factors and genetic polymorphisms of SOX11. We conducted further analysis on 1640 smokers who had SOX11 polymorphic rs77996007, rs66465560, or rs68114586. Smokers who had a betel-quid-chewing habit and at least one C allele of rs77996007, one C allele of rs66465560, or one Del allele of rs68114586 had a 9.225- (95% CI: 6.610–12.874), 8.376- (95% CI: 5.735–12.234), or 8.653-fold (95% CI: 5.913–12.664) higher risk of oral cancer than did individuals with the WT gene who did not have a betel-quid-chewing habit. In light of the above results, we suggested that the SOX11 gene polymorphisms have an impact on oral cancer susceptibility in betel nut consumers.

### 2.4. Association between SOX11 Polymorphic Genotypes and Clinical Features of Oral Cancer

We investigated the role of SOX11 genetic polymorphisms in the clinical status of oral cancer. Table 4 shows the distribution frequency of clinical features, such as clinical stage, tumor size, lymph node or distant metastasis, and cell differentiation grade, and SOX11 genotype frequencies in patients with oral cancer. Compared with individuals who were homozygous for the WT allele of rs77996007, patients with at least one C allele of rs77996007 were significantly associated with larger tumor size after controlling for age, betel quid chewing, cigarette smoking, and alcohol drinking (AOR, 1.324; 95% CI, 1.047–1.674; *p* = 0.019). However, we did not observe a significant association between SOX11 genotype frequencies of rs77996007 and clinical stage, lymph node or distant metastasis, or cell differentiation grade.

### 2.5. Association between SOX11 mRNA Expression and Clinical Characteristics of HNSCC Tissues from the TCGA Database

**Figure 1:**
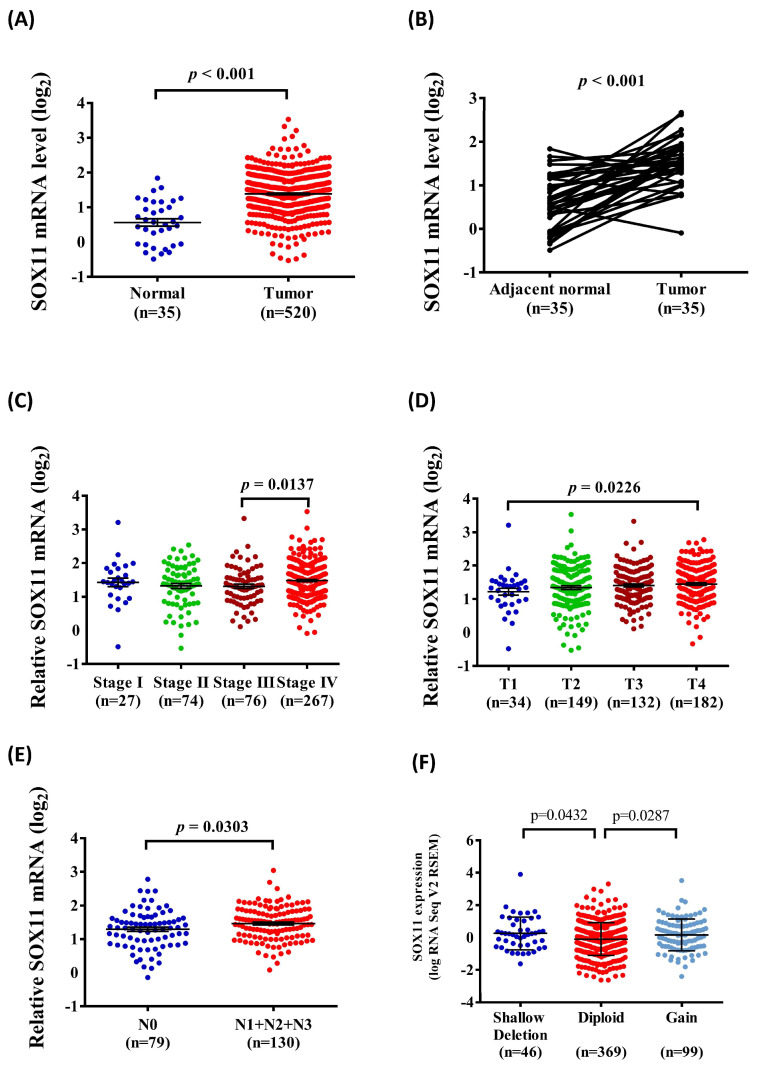
Association of SOX11 mRNA level and clinical status from the TCGA database. (**A**) The SOX11 mRNA level of HNSCC tissues were compared to normal tissues. (**B**) The SOX11 mRNA level were compared according to adjacent normal tissues and HNSCC tissues. (**C**) The SOX11 mRNA levels were compared according to tumor stage. (**D**) The SOX11 mRNA levels were compared according to tumor clinical T stage. (**E**) The SOX11 mRNA levels were compared according to clinical *n* stage. (**F**) Boxplots of RNA-Seq for SOX11 mRNA demonstrating significantly altered expression in diploid versus deletion and gain cancers.

Considering the potential effects of SOX11 polymorphic genotypes on SOX11 expression, we further clarified the clinical significance of SOX11 expression in HNSCC tissues from the TCGA database. As shown in Figure 1, the results indicated that SOX11 mRNA levels were upregulated in HNSCC compared with normal tissues (Figure 1A,B). Furthermore, the relative levels of SOX11 mRNA were significantly associated with the tumor status (Figure 1C), clinical T status (Figure 1D), and lymph node metastasis status (Figure 1E) in HNSCC tissues. Moreover, HNSCC samples with copy number loss express significantly higher levels of SOX11 mRNA than tumors diploid (*p* = 0.0432) (Figure 1F).

## 3. Discussion

SOX11 is involved in the processes of several human cancers, including bladder cancer, head and neck cancer and breast cancer. Previous studies have reported a direct association between tumor metastasis and *SOX11* expression [18,24,25,26]. However, the role of SOX11 polymorphisms in oral cancer has rarely been discussed. In the present study, we observed that the combined effect of environmental factors and *SOX11* polymorphisms considerably increased the risk of oral cancer. Moreover, patients with *SOX11* SNP rs77996007 with a genotype of TC and CC were significantly associated with large tumor size.

SNPs are among the most common types of genetic variation in the human genome. SNPs widely affect the regulation of genes that are associated with genetic susceptibility to cancer, such as those involved in cell cycle regulation, DNA mismatch repair, metabolism, and immunity [27,28,29]. Exploring the mechanisms through which SNPs affect cancer susceptibility is conducive to understanding the molecular pathogenesis of various cancers. The mechanism of SNPs can depend on their location within genes, such as promoters, exons, and introns as well as the 5′- and 3′-untranslated regions (UTRs) [30,31,32]. Promoter-associated polymorphisms affect the binding of transcription factors to alter promoter activity, further influencing gene transcription, mRNA stability, and translation [33,34].

The three SNPs of *SOX11* investigated in this study were localized in the 3′-UTR of the SOX11 gene. The 3′-UTR is a noncoding sequence that closely follows the coding regions of mRNA. The mRNA of nearly all eukaryotes involves 3′-UTR formation [35]; 3′-UTR formation is crucial for maintaining mRNA stability. Additionally, the 3′-UTR plays a major role in several aspects of gene regulation, including translation efficiency, subcellular localization, polyadenylation, and mRNA degradation [36,37]. Previous research revealed that the allelic variants of the *SOX11* SNPs in the 3′-UTR may modulate *SOX11* expression [38]. These data suggest that *SOX11* SNPs in the 3′-UTR have potential transcriptional function. The 3′-UTR can be bound by specific microRNAs (miRNAs), which can inhibit the translation of mRNAs or degrade mRNAs to inhibit their expression [39]. However, the three *SOX11* SNPs examined in the present study were determined to be in the 3′-UTR in which miRNAs may interact; therefore, they should be further delineated.

The present study is the first to explore the association between *SOX11* SNPs and oral cancer. We revealed the importance of *SOX11* variations in the development of oral cancer. However, this study has some limitations. Because the study included only the discovered population and there was no independent study to confirm these findings, our findings regarding the association between the *SOX11* variant and oral cancer should be considered preliminary. Moreover, we failed to rule out the possibility of potential selection bias, because the control group was selected from patients without cancer on a hospital basis. In addition, the mechanism through which *SOX11* SNPs regulate the development of oral cancer still requires further investigation.

## 4. Materials and Methods 

### 4.1. Study Population

Due to the unique epidemiology of oral cancer in Taiwan (with a ratio of almost 10:1 for men and women), this case–control study included 1196 male patients with oral cancer who underwent diagnosis at Changhua Christian Hospital in Changhua and Chung Shan Medical University Hospital in Taichung, Taiwan, between 2007 and 2019. For the control group, we enrolled 1200 healthy male participants aged >20 years and without a self-reported history of cancer at any site from Taiwan Biobank. In addition, subjects with oral precancerous disease such as leukoplakia, erythroplakia, etc. were excluded from the control group. Medical information—including primary tumor size, lymph node involvement, histologic grade, and pathologic stage, as determined according to the tumor–node–metastasis (TNM) staging system of the American Joint Committee on Cancer—was obtained from medical records [40]. Participants’ personal characteristics and information, including demographic characteristics, betel quid chewing, tobacco smoking, alcohol consumption, and medical histories, were obtained using interviewer-administered questionnaires, and informed written consent was obtained from each individual.

### 4.2. Determination of Genotypes

Genomic DNA from peripheral blood leukocytes was extracted using QIAamp DNA blood mini kits (Qiagen, Valencia, CA, USA) according to the manufacturer’s instructions as previously described [41,42]. DNA was quantified by measuring absorbance at 260 nm and stored at −20°C. Allelic discrimination for *SOX11* (rs77996007, rs66465560, and rs68114586) was performed using the TaqMan SNP genotyping assay (Applied Biosystems, Foster City, CA, USA), and further analysis was performed using SDS 3.0 software (Applied Biosystems).

### 4.3. Selection of SOX11 Polymorphisms and Database Analysis

In the dbSNP database, over 100 SOX11 SNPs have been documented. We investigated SOX11 (rs77996007, rs66465560, and rs68114586), those with minor allele frequencies ≥5% and located at the 3′UTR regions in the Chinese populations. For the database analysis, the association between SOX11 expression and clinical information of head and neck squamous cell carcinoma (HNSCC) tissues obtained from the Cancer Genome Atlas (TCGA) database was analyzed using GraphPad Prism 6 software (GraphPad Software Inc., La Jolla, San Jose, CA, USA). 

### 4.4. Statistical Analysis

Fisher’s exact test was used to determine differences in demographic characteristic between healthy controls and oral cancer patients. Adjusted odds ratios (ORs) along with the corresponding 95% confidence intervals (CIs) for the association between genotype frequencies and the risk of oral cancer plus clinicopathological characteristics were estimated using multiple logistic regression models after other covariates were controlled for. Data were analyzed using SAS 9.1 statistical software (SAS Institute Inc., Cary, NC, USA). A *p*-value of < 0.05 was considered significant.

## 5. Conclusions

Our results indicate that the allelic effects of SOX11 SNPs increase the risk of oral cancer based on environmental factors, such as tobacco smoking and betel quid chewing. Moreover, in oral cancer patients, SOX11 rs77996007 variants were significantly associated with large tumor size. Our results suggest that SOX11 rs77996007 is involved in the tumor progression of oral cancer.

## Figures and Tables

**Table 1:** The distributions of demographical characteristics in 1200 controls and 1196 male patients with oral cancer.

Variable	Controls (*n* = 1200)	Patients (*n* = 1196)	*p*-Value
Age (yrs)			
≦55	610 (50.8%)	609 (50.9%)	*p* = 0.966
>55	590 (49.2%)	587 (49.1%)	
Betel quid chewing			
No	1001 (83.4%)	321 (26.8%)	
Yes	199 (16.6%)	875 (73.2%)	*p* < 0.001 *
Cigarette smoking			
No	564 (47.0%)	192 (16.0%)	
Yes	636 (53.0%)	1004 (84.0%)	*p* < 0.001 *
Alcohol drinking			
No	963 (80.2%)	648 (54.2%)	
Yes	237 (19.8%)	548 (45.8%)	*p* < 0.001 *
Stage			
I + II		565 (47.2%)	
III + IV		631 (52.8%)	
Tumor T status			
T1 + T2		599 (50.1%)	
T3 + T4		597 (49.9%)	
Lymph node status			
N0		796 (66.6%)	
N1 + N2 + N3		400 (33.4%)	
Metastasis			
M0		1186 (99.2%)	
M1		10 (0.8%)	
Cell differentiation			
Well differentiated		170 (14.2%)	
Moderately or poorly differentiated		1026 (85.8%)	

Fisher’s exact test was used between healthy controls and patients with oral cancer. * *p*-value < 0.05 was considered statistically significant.

**Table 2:** The adjusted odds ratio (AOR) and 95% confidence interval (CI) of oral cancer associated with *SOX11* genotypic frequencies.

Variable	Controls (*n* = 1200) (%)	Patients (*n* = 1196) (%)	OR (95% CI)	AOR (95% CI) ^b^
**rs77996007**				
TT	514 (42.8%)	470 (39.3%)	1.000 (reference)	1.000 (reference)
TC	526 (43.8%)	586 (49.0%)	1.218 (1.026–1.446) ^a^	1.076 (0.871–1.328)
CC	160 (13.4%)	140 (11.7%)	0.957 (0.739–1.240)	0.923 (0.670–1.271)
TC + CC	686 (57.2%)	726 (60.7%)	1.157 (0.983–1.362)	1.042 (0.853–1.272)
**rs66465560**				
TT	911 (75.9%)	921 (77.0%)	1.000 (reference)	1.000 (reference)
TC	271 (22.6%)	265 (22.2%)	0.967 (0.798–1.173)	0.965 (0.762–1.223)
CC	18 (1.5%)	10 (0.8%)	0.550 (0.252–1.197)	0.424 (0.163–1.099)
TC + CC	289 (24.1%)	275 (23.0%)	0.941 (0.779–1.137)	0.929 (0.736–1.171)
**rs68114586**				
Ins/Ins	912 (76.0%)	921 (77.0%)	1.000 (reference)	1.000 (reference)
Ins/Del	269 (22.4%)	264 (22.1%)	0.972 (0.801–1.179)	0.978 (0.772–1.240)
Del/Del	19 (1.6%)	11 (0.9%)	0.573 (0.271–1.211)	0.435 (0.174–1.090)
Ins/Del or Del/Del	288 (24.0%)	275 (23.0%)	0.946 (0.783–1.142)	0.939 (0.745–1.184)

The odds ratio (OR) with their 95% confidence intervals were estimated by logistic regression models. ^a^ *p* = 0.024. ^b^ The adjusted odds ratio (AOR) with their 95% confidence intervals were estimated by multiple logistic regression models after controlling for age, betel quid chewing, cigarette smoking, and alcohol drinking.

**Table 3:** Associations of the combined effect of *SOX11* gene polymorphisms and betel nut chewing with the susceptibility to oral cancer among 1640 smokers.

Variable	Controls (*n* = 636) (%)	Patients (*n* = 1004) (%)	OR (95% CI)	AOR (95% CI)
**rs77996007**				
TT genotype & non-betel nut chewing	196 (30.8%)	86 (8.6%)	1.000 (reference)	1.000 (reference)
TC or CC genotype or betel nut chewing	330 (51.9%)	406 (40.4%)	2.804 (2.094–3.755)	2.623 (1.949–3.530)
TC or CC genotype with betel nut chewing	110 (17.3%)	512 (51.0%)	10.608 (7.652–14.705)	9.225 (6.610–12.874)
**rs66465560**				
TT genotype & non-betel nut chewing	336 (52.8%)	138 (13.8%)	1.000 (reference)	1.000 (reference)
TC or CC genotype or betel nut chewing	252 (39.6%)	678 (67.5%)	6.551 (5.126–8.372)	5.872 (4.575–7.535)
TC or CC genotype with betel nut chewing	48 (7.6%)	188 (18.7%)	9.536 (6.562–13.859)	8.376 (5.735–12.234)
**rs68114586**				
Ins/Ins genotype & non-betel nut chewing	338 (53.1%)	138 (13.8%)	1.000 (reference)	1.000 (reference)
Ins/Del or Del/Del genotype or betel nut chewing	251 (39.5%)	677 (67.4%)	6.606 (5.169–8.443)	5.934 (4.625–7.613)
Ins/Del or Del/Del genotype with betel nut chewing	47 (7.4%)	189 (18.8%)	9.849 (6.763–14.343)	8.653 (5.913–12.664)

The adjusted odds ratio (AOR) with their 95% confidence intervals were estimated by multiple logistic regression models after controlling for age and alcohol drinking.

**Table 4:** The adjusted odds ratio (AOR) and 95% confidence intervals (CI) of clinical statuses associated with genotypic frequencies of *SOX11* rs77996007 in male oral cancer patients (*n* = 1196).

Variable			AOR (95% CI)	*p*-Value
	**Clinical Stage**			
**rs77996007**	Stage I + II	Stage III + IV		
	(*n* = 565) (%)	(*n* = 631) (%)		
TT	238 (42.1%)	232 (36.8%)	1.00	
TC + CC	327 (57.9%)	399 (63.2%)	1.252 (0.991–1.582)	*p* = 0.060
	**Tumor size**			
**rs77996007**	≦T2	>Τ2		
	(*n* = 599) (%)	(*n* = 597) (%)		
TT	255 (42.6%)	215 (36.0%)	1.00	
TC + CC	344 (57.4%)	382 (64.0%)	1.324 (1.047–1.674)	*p* = 0.019 *
	**Lymph node metastasis**			
**rs77996007**	No	Yes		
	(*n* = 796) (%)	(*n* = 400) (%)		
TT	313 (39.3%)	157 (39.3%)	1.00	
TC + CC	483 (60.7%)	243 (60.7%)	1.000 (0.780–1.282)	*p* = 0.999
	**Distant metastasis**			
**rs77996007**	M0	M1		
	(*n* = 1034) (%)	(*n* = 10) (%)		
TT	468 (39.5%)	2 (20.0%)	1.00	
TC + CC	718 (60.5%)	8 (80.0%)	2.645 (0.555–12.597)	*p* = 0.222
	**Cell differentiation grade**			
**rs77996007**	≦Grade I (*n* = 170) (%)	>Grade I (*n* = 1026) (%)		
TT	63 (37.1%)	407 (39.7%)	1.00	
TC + CC	107 (62.9%)	619 (60.3%)	0.889 (0.634–1.246)	*p* = 0.494

Cell differentiate grade: grade I: well differentiated; grade II: moderately differentiated; grade III: poorly differentiated. The adjusted odds ratio (AOR) with their 95% confidence intervals were estimated by multiple logistic regression models after controlling for age, betel quid chewing, cigarette smoking, and alcohol drinking. * *p*-value < 0.05 as statistically significant.

## Appendix A

**Table A1:** The odds ratio (OR) and adjusted odds ratio (AOR) and 95% confidence interval (CI) of oral cancer associated with *SOX11* genotypic frequencies.

Variable	Controls (*n* = 1200) (%)	Patients (*n* = 1196) (%)	OR (95% CI)	*p* Value	AOR (95% CI)	*p* Value
**Age (yrs)**						
≦55	610 (50.8%)	609 (50.9%)	1.000 (reference)	*p* = 0.966	1.000 (reference)	*p* = 0.014
>55	590 (49.2%)	587 (49.1%)	0.997 (0.849–1.170)		1.283 (1.053–1.564)	
**Betel quid chewing**						
No	1001 (83.4%)	321 (26.8%)	1.000 (reference)	*p* < 0.001	1.000 (reference)	*p* < 0.001
Yes	199 (16.6%)	875 (73.2%)	13.711 (11.240–16.726)		10.708 (8.539–13.428)	
**Cigarette smoking**						
No	564 (47.0%)	192 (16.0%)	1.000 (reference)	*p* < 0.001	1.000 (reference)	*p* = 0.006
Yes	636 (53.0%)	1004 (84.0%)	4.637 (3.829–5.616)		1.398 (1.103–1.773)	
**Alcohol drinking**						
No	963 (80.2%)	648 (54.2%)	1.000 (reference)	*p* < 0.001	1.000 (reference)	*p* < 0.001
Yes	237 (19.8%)	548 (45.8%)	3.436 (2.864–4.122)		1.499 (1.197–1.877)	
**rs77996007**						
TT	514 (42.8%)	470 (39.3%)	1.000 (reference)	*p* = 0.079	1.000 (reference)	*p* = 0.746
TC + CC	686 (57.2%)	726 (60.7%)	1.157 (0.983–1.362)		1.034 (0.844–1.268)	
**rs66465560**						
TT	911 (75.9%)	921 (77.0%)	1.000 (reference)	*p* = 0.529	1.000 (reference)	*p* = 0.472
TC + CC	289 (24.1%)	275 (23.0%)	0.941 (0.779–1.137)		0.572 (0.125–2.619)	
**rs68114586**						
Ins/Ins	912 (76.0%)	921 (77.0%)	1.000 (reference)	*p* = 0.561	1.000 (reference)	*p* = 0.513
Ins/Del or Del/Del	288 (24.0%)	275 (23.0%)	0.946 (0.783–1.142)		1.665 (0.362–7.650)	

The adjusted odds ratio (AOR) with their 95% confidence intervals were estimated by multiple logistic regression models after controlling for age, betel quid chewing, cigarette smoking, and alcohol drinking.
